# Reconfigurable nonlinear Pancharatnam-Berry diffractive optics with photopatterned ferroelectric nematics

**DOI:** 10.1038/s41377-025-01981-0

**Published:** 2025-09-10

**Authors:** Hui-Feng Chen, Xin-Yu Tao, Bo-Han Zhu, Jin-Tao Pan, Ling-Ling Ma, Chao Chen, Wen-Guo Zhu, Wei Chen, Yan-Qing Lu

**Affiliations:** 1 National Laboratory of Solid State Microstructures, Key Laboratory of Intelligent Optical Sensing and Manipulation, College of Engineering and Applied Sciences, Nanjing University, 210023 Nanjing, China; 2 Research Institute of Superconductor Electronics (RISE), School of Electronic Science and Engineering, Nanjing University, 210023 Nanjing, China; 3 Key Laboratory of Optoelectronic Information and Sensing Technologies of Guangdong Higher Education Institutes, Department of Optoelectronic Engineering, Jinan University, 510632 Guangzhou, China

**Keywords:** Liquid crystals, Nonlinear optics

## Abstract

Planar optical elements incorporating space-varying Pancharatnam-Berry phase have revolutionized the manipulation of light fields by enabling continuous control over amplitude, phase, and polarization. While previous research focusing on linear functionalities using apolar liquid crystals (LCs) has attracted much attention, extending this concept to the nonlinear regime offers unprecedented opportunities for advanced optical processing. Here, we demonstrate the reconfigurable nonlinear Pancharatnam-Berry LC diffractive optics in photopatterned ion-doped ferroelectric nematics. By customizing the spatial phase distribution of efficient second-harmonic excitation, we accomplish programmable beam steering of various optical states towards predefined diffraction directions. Experimental results reveal continuous evolution of diffraction orders, intensity distributions, and polarization states under electrically varying splay conditions, consistent with our theoretical predictions. This work opens new avenues for designing reconfigurable nonlinear beam shaping and steering devices with potential applications in advanced optical and quantum information processing.

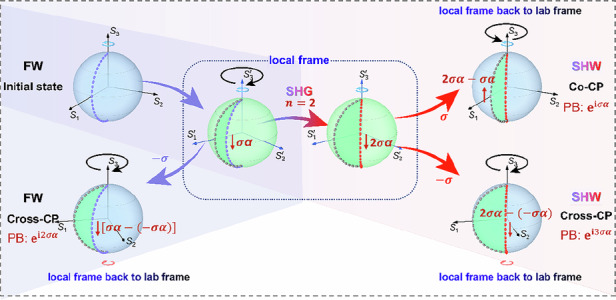

## Abstract (web summary)

Reconfigurable nonlinear Pancharatnam-Berry liquid crystals have been proposed, serving as a design framework for developing multifunctional dynamic nonlinear optics devices.

## Introduction

Planar optical elements with spatially variant Pancharatnam-Berry phases, especially LC-^1–7^ and metasurface-based^8–10^ components, have attracted considerable interest owing to their exceptional ability to continuously manipulate the amplitude, phase, and polarization of light fields, facilitating numerous exciting advancements in both scientific research and technological applications^11–13^. Unlike metasurfaces, which usually suffer from sophisticated fabrication processes and fixed properties once built, LC optical elements offer a unique combination of flexibility and ease of fabrication^14,15^. LCs leverage their self-assembling properties to form complex microstructures with diverse forms and tunability^16–18^. The versatility of LC systems is further highlighted by their ability to be fabricated through straightforward methods such as direct deposition onto a substrate or encapsulation within cells with predefined alignment patterns. These patterns can be established through photomasks^19^, photopatterning^20–22^, laser writing^23^, or others^24^. Significant advancements have been achieved in the development of high-performance polarization gratings^20^, Fresnel lenses^25,26^, versatile holograms^27–29^, etc^12,30,31^. By employing chiral LC, it is possible to achieve sub-wavelength grating periods through straightforward fabrication methods, as demonstrated in previous studies^32^, and enable the orthogonal modulation of multiple degrees of freedom of light^18,33^. However, all the above-mentioned research has predominantly concentrated on linear optical functionalities typically realized using apolar LC materials^34,35^. It is highly desirable to extend the concept of “planar LC optics”^4^ to the nonlinear case, leading to the development of nonlinear planar LC optics with embedded features capable of fully controlling the wavefront of the nonlinear output. This shift from linear to nonlinear optics offers opportunities for designing more innovative and unique functionalities, while preserving the intrinsic benefits of planar LC optics, including cost-effectiveness, lightweight properties, ease of fabrication, and flexibility^36,37^.

The discovery of ferroelectric nematic LCs (FNLCs) marks a major breakthrough in the field of soft matter research^38–40^. These materials combine LC properties with ferroelectricity, allowing them to flexibly pattern and tune their structure in response to external stimuli, which has spurred numerous subsequent studies focusing on the properties^41^, structures^42,43^, topologies^44^, formation mechanisms^45,46^, and identification of new states of matter in these fluids^47,48^, such as helielectric^49^, polar heliconical nematic^48^, and various new polar smectic phases^45,47^. Besides being highly intriguing from the fundamental point of view, these materials are also considered to hold great promise as a tunable soft material platform for nonlinear photonics^50,51^. Studies on the effectiveness of optical second-harmonic generation (SHG) in FN materials have demonstrated nonlinear susceptibilities^51,52^ of 5.6 pm·V^-1^ to 25 pm·V^-1^—comparable to those of solid-state nonlinear crystals—highlighting their potential to become optimal candidates for the next-generation nonlinear optical devices^51^. Specifically structured FNLC materials with continuously helielectric polar configurations have shown nontrivial phase matching, leading to largely improved second-order nonlinear optical response^53^. More recently, our group proposed the nonlinear Pancharatnam-Berry phase in the FNLC film, associated with cascaded linear and nonlinear optical spin-orbit interactions, for multi-channel linear and nonlinear optical vortex generation^54^. However, the real-time reconfigurable tuning of the nonlinear Pancharatnam-Berry phase remains elusive.

Here, we transform the Pancharatnam-Berry LC optics to the reconfigurable nonlinear Pancharatnam-Berry LC optics regime by employing photopatterned, electrically-controlled, ion-doped FNLCs. We map the diffracted optical fields under different splay conditions of material polarizations and various pumping light polarizations. Leveraging the in-plane electric field, we demonstrate customized tunable beam steering of different optical states into predefined diffraction directions with continuously evolved diffraction orders, intensity distributions, and carrying polarizations. These experimental results, which align well with theoretical predictions, serve as a design framework for developing dynamic nonlinear optical devices tailored for specific beam shaping and steering applications. This work opens new avenues for next-generation reconfigurable nonlinear photonic devices, with promising applications in advanced optical processing and quantum information technologies.

## Results

### Nonlinear Pancharatnam-Berry LCs

**Fig. 1:**
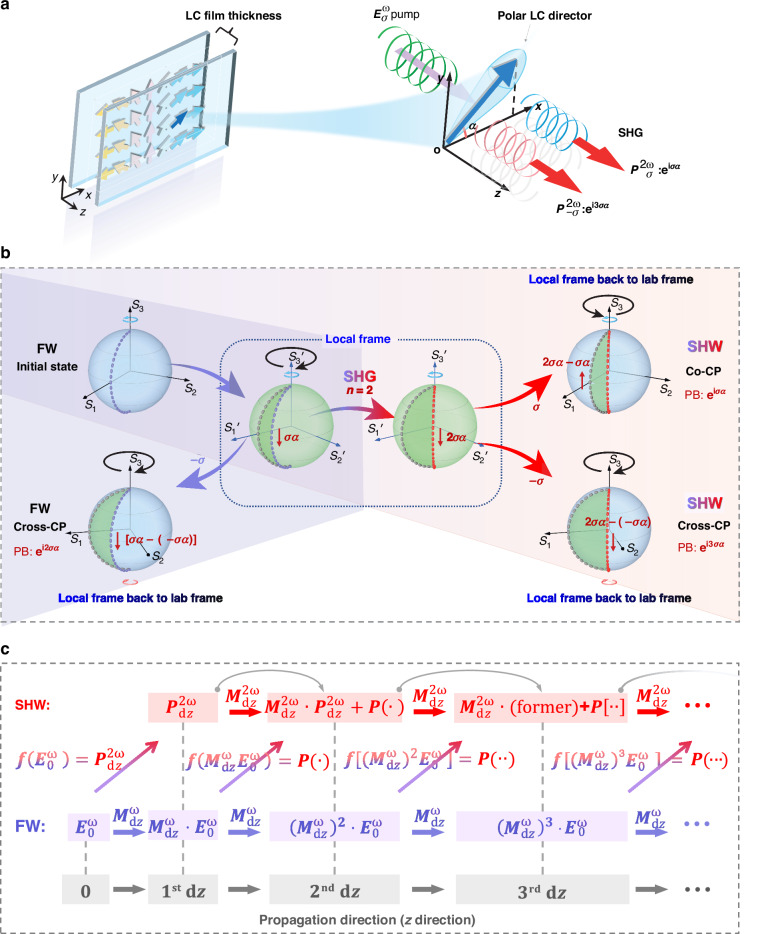
**Nonlinear Pancharatnam-Berry phase.** **a** A thin-film polar LC element with a spatially modulated alignment pattern in the *x*-*y* plane. The polar LC molecules are sandwiched between two photopatterned substrates. The right panel: a polar LC director in the (*x, y, z*) coordinate system. The azimuthal angle in the *x-y* plane is denoted as $\alpha$. For a polar LC director, SHG signals of both circular polarizations, with phases of *σα* and 3*σα*, are generated in the forward direction. The incident fundamental wave (FW) is at *ω* and the generated second-harmonic wave (SHW) is at 2*ω*. **b** Schematic representation of the linear and nonlinear Pancharatnam-Berry phases resulting from coordinate transformations. The entire diagram is divided into a central part representing the local frame (*S*_1_′, *S*_2_′, *S*_3_′) with blue coordinate axes and an outer part representing the laboratory coordinates (*S*_1_, *S*_2_, *S*_3_) with black coordinate axes. The area under the blue-violet trapezoidal shadow on the left corresponds to the linear optical process, while the other gradient-colored trapezoidal region includes the process of nonlinear frequency conversion. For a CP FW incidence with the spin state of *σ* = +1 (RHCP), the evolved cross-CP FW signal acquires Pancharatnam-Berry phase of 2*σα*, while the co- and cross-CP components of the SH signal acquire nonlinear Pancharatnam-Berry phases *σα* and 3*σα*, respectively. The blue and red circumferential arrows above or below the Poincaré sphere represent the polarization state in the current configuration. The blue ones correspond to the RHCP, and the red ones correspond to the LHCP. The black circumferential arrows correspond to the rotation direction of the Poincaré sphere. The unidirectional arrows and their colors between Poincaré spheres denote the process within the framework of linear optics or nonlinear optics. The dark red arrows adjacent to the evolution path represent the direction of polarization evolution. The gray, blue-violet, and red dashed curves are used to indicate the angle relative to the *α* = 0 geodesic passing through *S*_1_′. Specifically, the gray dashed line denotes the reference line passing through *S*_1_′; the blue-violet and red dashed curves represent the geodesic trajectories of FW and SHW spans certain degrees with the *α* = 0 geodesic (gray dashed curve). **c** Schematic of the cascaded linear and nonlinear spin-coupling processes. ${\rm{d}}z$: unit thickness of each infinitesimally thin LC layer. ${{\boldsymbol{E}}}_{0}^{{\rm{\omega }}}$: incident FW. ${{\boldsymbol{P}}}_{{\rm{d}}z}^{2{\rm{\omega }}}$: SHW excited from the 1^st^ LC layer. ${{\boldsymbol{M}}}_{{\rm{d}}z}^{{\rm{\omega }}}$ and ${{\boldsymbol{M}}}_{{\rm{d}}z}^{2{\rm{\omega }}}$: Jones matrices describing the linear optical processes of FW and SHW following propagation through a distance d*z*, respectively. The function ${\boldsymbol{f}}\left(\cdot \right)$ is employed to mathematically represent the physical process of nonlinear frequency conversion

The concept of our proposed nonlinear Pancharatnam-Berry LCs is illustrated in Fig. 1a. A structured polar LC film is fabricated with a spatially modulated alignment pattern in the *x*-*y* plane through photopatterning. The thickness of the film is generally on the order of micrometers. For each LC pixel with a specific LC director, a mathematical concept used to describe the average orientation of LC molecules in this pixel, when illuminated by circularly polarized (CP) pump light propagating along the *z*-axis (*k // z*), the transmissive polar LC enables spin-dependent nonlinear Pancharatnam-Berry phase manipulation due to the spin-coupling effect.

An intuitive representation of the linear and nonlinear Pancharatnam-Berry phase shift, governed by the orientation angle *α* of the medium’s anisotropic structure and the spin state *σ* ($\sigma =\pm 1$ for right/left-handed circular polarization (RHCP and LHCP)) of the incident light, is illustrated in Fig. 1b. The entire diagram is divided into a central part representing the local frame and an outer part representing the laboratory coordinates. The area under the blue-violet trapezoidal shadow on the left corresponds to the linear optical process, while the other gradient-colored trapezoidal region includes the process of nonlinear frequency conversion.

In the linear optical process, we consider the interaction of a CP fundamental wave (FW) with a spin state of *σ* propagating through a very thin LC medium. The LC director is rotated by an angle *α* relative to the laboratory’s transverse coordinate. Suppose the incident light is prepared in a RHCP (*σ*, at the north pole of the Poincaré sphere) in the fundamental frequency ${\rm{\omega }}$, the lab frame Poincaré sphere is rotated about *S*_3_ by an angle *σα* to the local frame. In the local frame, the geodesic trajectory of the fundamental frequency (blue-violet dashed curve) spans an angle *σα* with the *α* = 0 geodesic passing through *S*_1_′ (gray dashed curve)^55^. As the light propagates through the LC medium, its polarization state evolves along a great circle on the Poincaré sphere, moving from the north pole towards the south pole, as indicated by the blue-violet dashed curve. In this scenario, the cross-CP component of the FW acquires a spin state of −*σ* upon exiting the LC medium. Then, the transformation of the Poincaré sphere back to the laboratory frame (in an inverse rotation direction) leads to a closed trajectory with an angle of *σα* – (–*σα*) = 2*σα* relative to the *S*_1_ axis, giving a Pancharatnam-Berry phase of 2*σα*^56,57^.

In the context of nonlinear optical processes, the interaction between FW and LCs involves simultaneous changes in both polarization state and frequency^55^. As previously established, within the local frame, the geodesic trajectory of the fundamental frequency spans an angle *σα* relative to *α* = 0 geodesic passing through *S*_1_′. Frequency conversion to the *n*-th harmonic (with *n* = 2 for SHG) amplifies this angular displacement by a factor of *n*, resulting in a new angle spanned relative to the *S*_1_′ axis, specifically *nσα*. The polarization state of the *n*-th harmonic is positioned at the south pole of the Poincaré sphere. At this stage, two possible outcomes emerge: (1) The polarization state may revert back to its initial CP configuration (*σ* → *σ*). In this case, the rotation of the Poincaré sphere back to the laboratory frame induces a closed trajectory with an angle of *nσα* – (*σα*) = (*n* – 1) *σα* relative to the *S*_1_ axis. For *n* = 2, this results in a nonlinear Pancharatnam-Berry phase of *σα*. (2) Conversely, the polarization state may remain in its converted CP state (*σ* → −*σ*). Here, the rotation back to the laboratory frame leads to a trajectory with an angle of *nσα* – (–*σα*) = (*n* + 1) *σα* relative to the *S*_1_ axis, yielding a nonlinear Pancharatnam-Berry phase of 3*σα* for *n* = 2. These findings demonstrate how the orientation angle *α* of LCs, in conjunction with the spin state *σ* and harmonic order *n*, influences the nonlinear Pancharatnam-Berry phase. Consequently, the nonlinearity phase imparted by polar LCs can be flexibly adjusted by modulating the orientation distribution of LC directors, paving the way for innovative applications in soft-matter photonics. Next, we present the analytical calculation of the excited nonlinear polarizations from a structured polar LC element.

FNLCs, also known as polar LCs with broken inversion symmetry, exhibit strong nonlinear optical responses characterized by a non-zero second-order nonlinear optical susceptibility. At 1064 nm, the components of this susceptibility are given by ${\chi }_{{aaa}}^{(2)}=11.2\,{\rm{pm}}\cdot {{\rm{V}}}^{-1}$ and ${\chi }_{{abb}}^{(2)}={\chi }_{{bab}}^{(2)}={\chi }_{{bba}}^{(2)}=1.2\,{\rm{pm}}\cdot {{\rm{V}}}^{-1}$, where the *a*- and *b*-axes are parallel and perpendicular to the polar LC director, respectively^51^. To derive the nonlinear Pancharatnam-Berry phase, we employ a CP basis $(L,R,z)$, where *L* and *R* denote the LHCP and RHCP, and *z* is the propagation direction. In addition, the second-order nonlinear susceptibility tensor of the LC medium is transformed to this CP basis as ${\chi }_{{lmn}}^{(2)}$, where $l,m,n=(L,R,z)$, thus allowing for the analysis of nonlinear polarization generation and its associated nonlinear Pancharatnam-Berry phase through coordinate transformations in the circular basis and the frequency-doubling process.

As illustrated in Fig. 1c, the polar LC film is divided into numerous infinitesimally thin layers along its thickness, with each layer having a thickness of d*z*. It is assumed that the orientation angle *α* remains unchanged across these layers. Notably, *α* can represent either a uniformly aligned anisotropic LCs or a specific director distribution corresponding to structured polar LC elements. Additionally, it is assumed that molecular interactions and resultant optical effects between adjacent LC pixels are negligible. For the *N*^th^ LC layer, the modulated FW at frequency *ω* is given by:

1 $${{{{\boldsymbol{E}}}_{N{\rm{d}}z}^{{\rm{\omega }}}=\left({{\boldsymbol{M}}}_{{\rm{d}}z}^{{\rm{\omega }}}\right)}^{N}\cdot {\boldsymbol{E}}}_{0}^{{\rm{\omega }}}$$

where ${{\boldsymbol{M}}}_{{\rm{d}}z}^{{\rm{\omega }}}$ represents the Jones matrix describing the linear optical process experienced by the FW as it propagates through a unit-thin LC layer. The nonlinear polarization generated at this layer is:

2 $${{\boldsymbol{P}}}_{N{\rm{d}}z}^{2{\rm{\omega }}}={\boldsymbol{f}}\left[{{\left({{\boldsymbol{M}}}_{{\rm{d}}z}^{{\rm{\omega }}}\right)}^{N-1}\cdot {\boldsymbol{E}}}_{0}^{{\rm{\omega }}}\right],\,(N\ge 1)$$

Here, ${\boldsymbol{f}}$ encapsulates the physical processes associated with nonlinear frequency conversion. For an arbitrary polarized FW incident at normal incidence: ${{\boldsymbol{E}}}^{{\rm{\omega }}}(0)={E}_{R}^{{\rm{\omega }}}(0){\hat{{\bf{e}}}}_{R}+{E}_{L}^{{\rm{\omega }}}(0){\hat{{\bf{e}}}}_{L}$, the nonlinear polarization can be decomposed into RH and LH components, denoted as ${P}_{R}^{2{\rm{\omega }}}(N{\rm{d}}z)$ and ${P}_{L}^{2{\rm{\omega }}}(N{\rm{d}}z)$, respectively. These components are expressed as:

3 $$\begin{array}{c}{P}_{R}^{2{\rm{\omega }}}(N{\rm{d}}z)={\varepsilon }_{0}\left[\begin{array}{c}{\chi }_{{R}^{{\prime} }{R}^{{\prime} }{R}^{{\prime} }}^{\left(2\right)}{{\rm{e}}}^{{\rm{i}}\alpha }{\left({E}_{R}^{{\rm{\omega }}}\left(N{\rm{d}}z\right)\right)}^{2}+{\chi }_{{R}^{{\prime} }{L}^{{\prime} }{L}^{{\prime} }}^{\left(2\right)}{{\rm{e}}}^{-{\rm{i}}3\alpha }{\left({E}_{L}^{{\rm{\omega }}}\left(N{\rm{d}}z\right)\right)}^{2}\\ +2{\chi }_{{R}^{{\prime} }{R}^{{\prime} }{L}^{{\prime} }}^{\left(2\right)}{{\rm{e}}}^{-{\rm{i}}\alpha }\left({E}_{R}^{\omega }\left(N{\rm{d}}z\right){E}_{L}^{\omega }\left(N{\rm{d}}z\right)\right)\end{array}\right]\\ {P}_{L}^{2{\rm{\omega }}}(N{\rm{d}}z)={\varepsilon }_{0}\left[\begin{array}{c}{\chi }_{{L}^{{\prime} }{R}^{{\prime} }{R}^{{\prime} }}^{\left(2\right)}{{\rm{e}}}^{{\rm{i}}3\alpha }{\left({E}_{R}^{{\rm{\omega }}}\left(N{\rm{d}}z\right)\right)}^{2}+{\chi }_{{L}^{{\prime} }{L}^{{\prime} }{L}^{{\prime} }}^{\left(2\right)}{{\rm{e}}}^{-{\rm{i}}\alpha }{\left({E}_{L}^{{\rm{\omega }}}\left(N{\rm{d}}z\right)\right)}^{2}\\ +2{\chi }_{{L}^{{\prime} }{R}^{{\prime} }{L}^{{\prime} }}^{\left(2\right)}{{\rm{e}}}^{{\rm{i}}\alpha }\left({E}_{R}^{{\rm{\omega }}}\left(N{\rm{d}}z\right){E}_{L}^{{\rm{\omega }}}\left(N{\rm{d}}z\right)\right)\end{array}\right]\end{array}$$

where ${E}_{R}^{{\rm{\omega }}}(N{\rm{d}}z)$ and ${E}_{L}^{{\rm{\omega }}}(N{\rm{d}}z)$ refer to the RHCP and LHCP components of the propagating FW at the *N*d*z* plane, and ${\chi }_{{R}^{{\prime} }{R}^{{\prime} }{R}^{{\prime} }}^{(2)}={\chi }_{{R}^{{\prime} }{L}^{{\prime} }{L}^{{\prime} }}^{(2)}={\chi }_{{R}^{{\prime} }{R}^{{\prime} }{L}^{{\prime} }}^{(2)}={\chi }_{{L}^{{\prime} }{L}^{{\prime} }{L}^{{\prime} }}^{(2)}={\chi }_{{L}^{{\prime} }{R}^{{\prime} }{R}^{{\prime} }}^{(2)}={\chi }_{{L}^{{\prime} }{R}^{{\prime} }{L}^{{\prime} }}^{(2)}=\frac{1}{2\sqrt{2}}{\chi }_{{aaa}}^{(2)}$. The derivation of these expressions is detailed in Supporting Information Text 1. The *z*-plane can be located either within the LC film ($z=N\cdot {\rm{d}}z < d$) or at its exit surface ($z=d$), where *d* denotes the total thickness of the LC film. Notably, in the aforementioned derivation, the dynamic processes associated with phase mismatch are not explicitly considered; instead, the analysis focuses exclusively on the evolution of the acquired geometric phase. As depicted in Fig. 1c, the generated SHW by the *N*^th^ LC layer will be superimposed onto the subsequent layers. This cumulative effect propagates through each layer, with the SHW generated at each step acting as the source for further propagation in the following layers. Ultimately, the total nonlinear polarization observed at the exit plane is the accumulation of contributions from all individual layers.

For a simplified scenario, an incident FW with RHCP evolves into two distinct components ${E}_{R}^{{\rm{\omega }}}(N{\rm{d}}z)\propto {E}_{R}^{{\rm{\omega }}}(0)$ and ${E}_{L}^{{\rm{\omega }}}(N{\rm{d}}z)\propto {{\rm{e}}}^{{\rm{i}}2\alpha }{E}_{R}^{{\rm{\omega }}}(0)$ upon propagation and reaching the *N*^th^ LC layer due to the linear spin-coupling effect. By substituting them into Eq. (3), the nonlinear polarization components at the *N*^th^ layer are derived as:

4 $$\begin{array}{c}{P}_{R}^{2{\rm{\omega }}}(N{\rm{d}}z)\propto {\varepsilon }_{0}{\chi }_{{R}^{{\prime} }{R}^{{\prime} }{R}^{{\prime} }}^{(2)}{{\rm{e}}}^{{\rm{i}}\alpha }{\left({E}_{R}^{{\rm{\omega }}}(N{\rm{d}}z)\right)}^{2}\\ {P}_{L}^{2{\rm{\omega }}}(N{\rm{d}}z)\propto {\varepsilon }_{0}{\chi }_{{L}^{{\prime} }{R}^{{\prime} }{R}^{{\prime} }}^{(2)}{{\rm{e}}}^{{\rm{i}}3\alpha }{\left({E}_{R}^{{\rm{\omega }}}(N{\rm{d}}z)\right)}^{2}\end{array}$$

This result demonstrates the emergence of distinct nonlinear Pancharatnam-Berry phases from the *N*^th^ LC layer for co- and cross-CP SHW components, specifically $\alpha$ and $3\alpha$, respectively. When these components propagate and reach the exit plane, we obtain $$\begin{array}{c}\ {P}_{R}^{2{\rm{\omega }}}(d)\propto {{\rm{e}}}^{{\rm{i}}\alpha }\\ \ {P}_{L}^{2{\rm{\omega }}}(d)\propto {{\rm{e}}}^{{\rm{i}}3\alpha }\end{array}$$ with spin-locked nonlinear Pancharatnam-Berry phases. Hence, it is evident that the final accumulation process does not alter the phase relationship inherently tied to the optical spin state. In summary, this simplified analysis highlights that the total nonlinear polarization exhibits the same form as described by Eq. (4), when neglecting dynamic processes. Such insights are fundamental for understanding and predicting nonlinear optical phenomena in structured polar LC systems.

### Nontrivial nonlinear diffractions

**Fig. 2:**
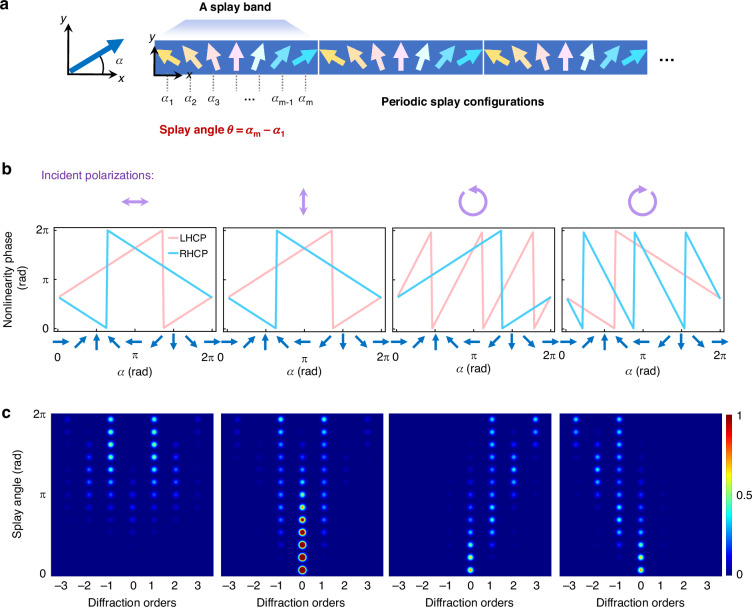
**Nonlinear diffraction simulations.** **a** Schematic of the orientations of polar LC directors in periodic splay bands. The splay angle illustrates the variation of the director vectors within a single period, which is defined as the difference between the minimum and maximum orientational angles within one period of the polar LC grating, i.e., the splay angle $\theta ={\alpha }_{m}-{\alpha }_{1}$. **b**, **c** Simulated nonlinearity phases and diffractions of SH signals for FW with horizontal polarization, vertical polarization, LHCP, and RHCP incidences, respectively. The nonlinearity phase describes the Pancharatnam-Berry phase generated from LCs with a specific director oriented at $\alpha$. The blue arrows located at the bottom of **b** represent the polar LC directors corresponding to different *α* angles and various nonlinearity phases. The diffraction patterns in (**c**) map the diffractions of SH signals under different conditions, including different polar LC gratings with distinct splay angles and different incident polarizations

Based on the above principle, we provide simulated nonlinearity phases and diffractions based on different polar LC gratings to direct the design of nonlinear beam steering devices (Fig. 2). The period of these gratings is ~50 µm. Within each period, the orientations of polar LC directors exhibit a continuous gradient, adopting a splay arrangement (Fig. 2a). This configuration ensures the molecular distribution within each period is symmetrically aligned relative to the grating stripes. These periodic configurations enable control over the optical properties of the grating, facilitating applications in advanced beam manipulation and nonlinear photonics.

Figure 2b presents a detailed analysis of the calculated nonlinearity phases as a function of LC director orientation $\alpha$, along with their corresponding spatial intensity distributions of SH signals generated by the proposed polar LC gratings. The investigation encompasses four sets of results for different incident polarization states and examines the diffraction behavior across a range of splay angles (0 to $2{\rm{\pi }}$). Under linearly polarized FW excitation, the LHCP and RHCP SHW components exhibit axisymmetric phase gradients with identical slopes in magnitude as the variation in *α*. This behavior is consistent with theoretical predictions in Eq. (1), confirming the validity of the proposed model. Concomitantly, the diffraction patterns of the SH signals display symmetric intensity distributions across the ± diffraction spots. When excited with CP FW, simulations reveal that the nonlinear phase of co-CP components is threefold that of cross-CP components. The above nonlinearity phase analysis is important for designing nonlinear photonic devices for beam shaping and steering.

Furthermore, we simulate the tailorable spatial intensity distribution of SH signals by adjusting the splay angle of polar LCs within a period (Fig. 2c and Fig. S1; Supporting Information Text 1). This modulation enables continuous control over both the diffraction orders and the intensity of SH signals. For instance, under RHCP pump light, the SH signal’s diffraction orders are locked to the +1-st and +3-rd orders at a splay angle of $2{\rm{\pi }}$. As the splay angle decreases, the intensity of the +1-st diffraction order follows a trend of decreasing, then increasing, and subsequently decreasing again, with its maximum occurring near $\theta =2{\rm{\pi }}/3$. Simultaneously, the intensity of the +3-rd diffraction order gradually diminishes. The intensity of the +2-nd diffraction order initially increases, then decreases, reaching its maximum near $\theta =4{\rm{\pi }}/3$. When the splay angle further decreases towards zero, the nonlinear Pancharatnam-Berry phase gradually disappears, resulting in the cessation of diffraction and a concentration of intensity at the zeroth order. Another example involves horizontally polarized light incidence. As the splay angle decreases from $2{\rm{\pi }}$ to 0, the maximum intensities of different diffraction orders (zero-th, ±1-st, ±2-nd, ±3-rd) occur at specific angles of $5/6{\rm{\pi }}$, $5/3{\rm{\pi }}$, $5/4{\rm{\pi }}$, $2{\rm{\pi }}$ (Fig. S2). These simulation results provide an intuitive understanding of the intricate interplay between the nonlinear Pancharatnam-Berry phase, splay angle, incident polarization, and SH signal distribution, offering insights into the continuous tuning of both nonlinearity phases and SH beam steering.

**Fig. 3:**
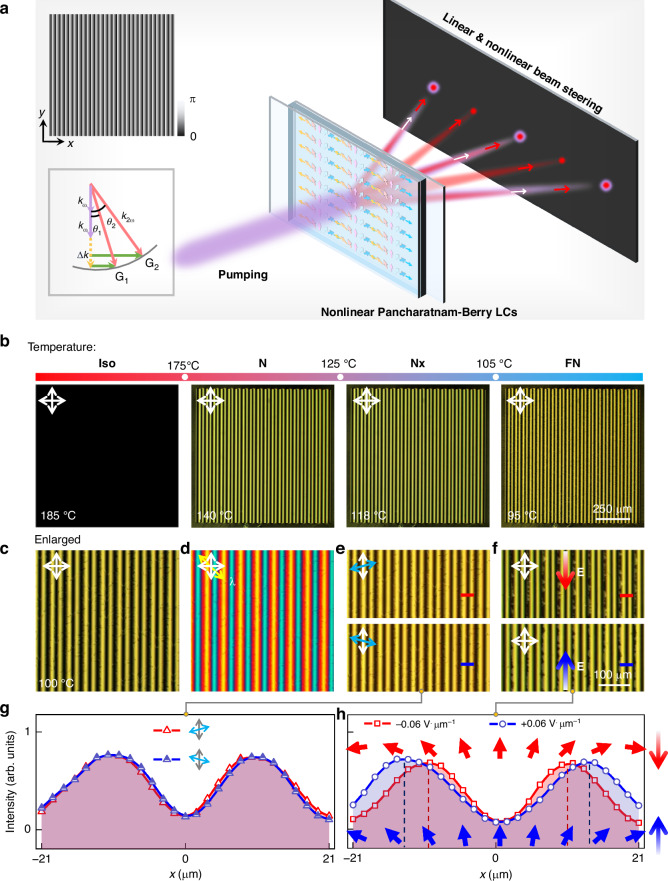
**Gradient nonlinear Pancharatnam-Berry LC devices.** **a** Scheme of linear and nonlinear optical modulation based on the fabricated FNLC device with a splay angle of ${\rm{\pi }}$. The upper-left inset schematically depicts the alignment configuration of the system, while the lower-left inset illustrates the nonlinear Raman-Nath diffraction mechanism, which facilitates transverse phase matching through periodic modulation of nonlinear susceptibility. In the right panel, the red and white arrows correspond to the propagations of SHW and FW, respectively. **b** Polarizing optical microscope images of defect-free gradient polarity patterns of ion-doped FNLCs during the thermal annealing process. Iso: isotropic phase; N: nematic phase; Nx: mesophase; Scale bar: 250 μm. **c**–**e** Polarizing optical microscope characterizations of the polar LC arrangement in the device. These images include (c) an enlarged texture, (d) an image under the cross-polarizing optical microscope with a full wave plate insertion, **e** two images of rotating the analyzer in opposite directions with the included angles of 75° and 105° between the polarizer and analyzer. **f** Polarizing optical microscope textures under an in-plane triangular-wave electric field (1 Hz, AC voltage 0.06 V·μm^-1^). The red and blue unidirectional arrows indicate the direction of the electric field. Scale bar: 100 μm. **g**, **h** Extracted intensity profiles from the textures in (**e**) and (**f**). Red and blue sets of arrows in (h) denote the corresponding orientations of polar LC directors

To experimentally demonstrate the nonlinear Raman-Nath diffractive steering, FNLC devices are synthesized, which consist of two glass substrates, both coated with photoalignment layers that are photopatterned to guide the arrangement of polar LC molecules. As shown in the upper-left inset of Fig. 3a, the alignment configuration is defined by $\alpha \left(x,{y}\right)=\frac{{\rm{\pi }}[L-{\mathrm{mod}}(x,L)]}{L}$, where *L* represents the period of the pattern, i.e., the alignments are continuously varied with a splay angle $\theta ={\rm{\pi }}$, as a primary demonstration (Sample A). Following the infiltration of ion-doped polar LCs, a gradient polar LC grating is formed with periodic splay configurations. It is worth noting that the use of 0.9% BMIM-PF6 ion-doped FNLC and a thermal annealing process at a rate of 2 °C per minute facilitate the optimal assembly of spontaneous polarization, promoting large-area and defect-free periodic patterns (Fig. 3b), in contrast to undoped FNLC patterns (Fig. S3). The underlying polar LC director structure is deduced through polarizing optical microscopy (POM) examinations (Fig. 3b–e) and the application of an in-plane electric field (Fig. 3f). Specifically, Fig. 3b presents the texture changes during cooling under a crossed POM, revealing large-scale smoothly varying gratings that remain stable down to ~70 °C. The enlarged POM texture (Fig. 3c) and the POM texture with a full wave plate at 135° (Fig. 3d) further demonstrate the desired director distribution that is determined by the anchoring. Periodic extinctions observed in Fig. 3c and POM textures between de-crossed polarizers (Fig. 3e) confirm the uniformity of LC directors across the cell thickness direction, which suppresses the twists, consistent with previously reported work^41^. Due to the flexoelectric coupling effect^42^, the overall director and polarization distributions are schematically represented in Fig. 3a. To verify this proposed structure, we apply an in-plane DC electric field to the sample (Supplementary Video 1). When ${\boldsymbol{E}}=(\mathrm{0,1,0})$, the polar LC director tends to align with the electric field, resulting in a decrease in the splay angle. This implies that within a splay band, the region aligned with the electric field occupies a larger spatial extent, consistent with the observed broadening of extinction regions (Fig. 3f, bottom panel; Fig. 3h, blue curve). Conversely, reversing the field direction to ${\boldsymbol{E}}=(0,-1,0)$ increases the splay angle, leading to narrower extinction regions (Fig. 3f, upper panel; Fig. 3h, red curve). These experimental results strongly support the proposed polar structure depicted in Fig. 3a. By analyzing the intensity variations along the grating vector within a period, we deduce that the splay angle variation is about $3/8{\rm{\pi }}$ (Fig. S4).

**Fig. 4:**
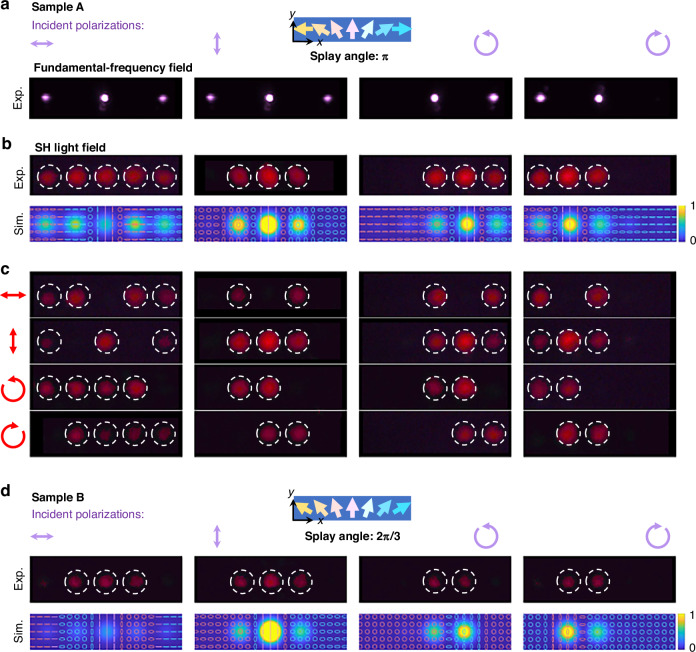
**Nonlinear diffractions.** **a** Diffraction patterns of the FW under illumination with horizontally polarized, vertically polarized, LHCP, and RHCP light. **b** Simulated and experimental nonlinear diffraction patterns of the FNLC device with a splay angle of ${\rm{\pi }}$. The corresponding local polarization states are also plotted in simulated SH fields, with the red and blue colors representing different spins. **c** Analyzed diffraction patterns by a polarizer and the combination of a polarizer and a 1/4 waveplate. **d** Simulated and experimental nonlinear diffraction patterns of the FNLC device with a splay angle of $2{\rm{\pi }}/3$. The colored unidirectional arrows in (**a**) and (**d**) illustrate the orientations of polar LCs

**Fig. 5:**
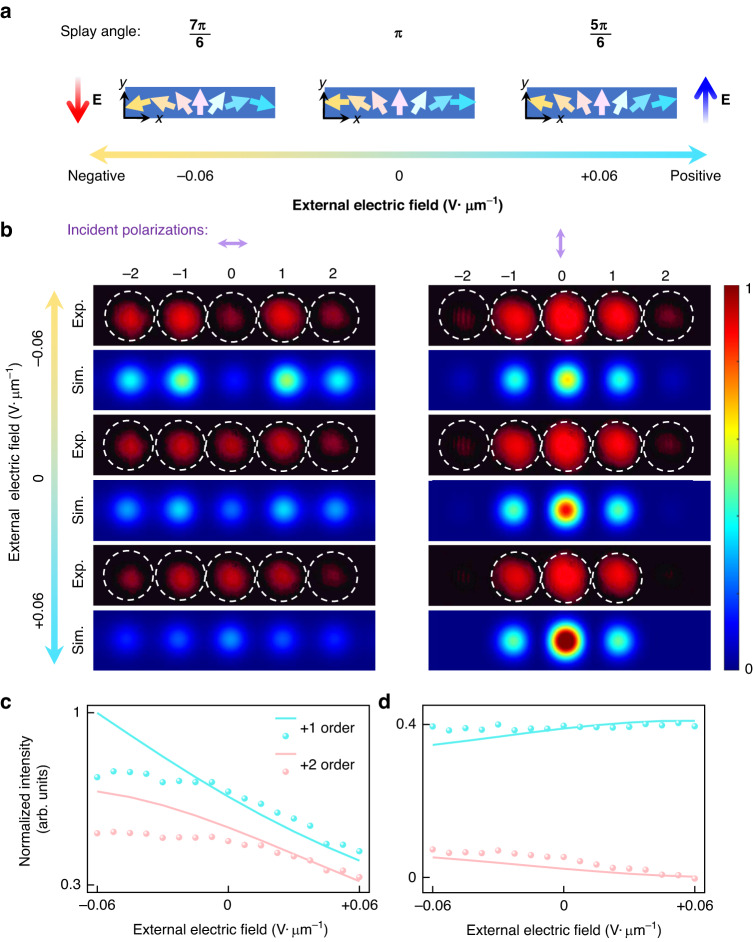
**Dynamical tuning of nonlinear diffraction.** **a** Demonstration of the dynamic modulation of the splay angle through the application of opposite-direction in-plane electric fields along the grating stripe direction. The variation in the splay angle is estimated to reach a maximum of ±${\rm{\pi }}/6$ at the maximum electric field strength of ±0.06 V·μm^-1^ (attributed to instrumental range limitations). **b** Simulated and experimental diffraction patterns of SH signals under illumination with horizontally polarized and vertically polarized light. **c**, **d** Simulated and experimental intensity changes for the +1-st and +2-nd orders of SH signals as the electric field increases from −0.06 V·μm^-1^ to +0.06 V·μm^-1^. The incident polarization is horizontal polarization for (**c**) and vertical polarization for (**d**)

Then, we employ the periodic splay FNLC patterns as transmissive planar optical elements to demonstrate both linear and nonlinear optical modulation processes (Figs. 4 and 5). In the N phase, **Sample A** exhibits a spatially homogeneous orientation of polar LCs ($\theta ={\rm{\pi }}$), functioning as a cycloidal diffractive waveplate, also referred to as a polarization grating in linear optics. Under linearly polarized incidence, the diffraction pattern in the linear optics regime consists of the zeroth-order and ±1-st orders. For CP light, only the zeroth-order and either the +1- or −1-st orders are observed, depending on the incident spin state. These linear optical effects are consistent with theoretical predictions for polarization gratings, which are sensitive to the half-wave condition.

As the temperature decreases to 90 °C, the sample transforms into the ferroelectric nematic phase. We illuminate it with femtosecond laser pulses at 1300 nm and measure the SHG signals using a charge-coupled device camera. Figure 4b presents the SHG diffraction patterns excited by horizontally polarized, vertically polarized, LHCP, and RHCP FWs. In this case, the phase-matching condition is satisfied transversely (perpendicular to the propagation direction) via momentum compensation provided by the grating vector of the modulated LC structure rather than relying on conventional longitudinal phase matching (inset in Fig. 3a). This mechanism enables efficient energy transfer between diffracted orders while circumventing stringent phase-velocity matching requirements typically associated with nonlinear optical processes. When incident light is horizontally polarized, the generated SHG signals include five distinct diffraction orders. Cross-CP components manifest at the ±2-nd diffraction orders, while linear polarization states (along the *x* and *y* axes) appear at the ±1-st and zeroth orders. Conversely, for vertically polarized incidence, the intensities at the ±2-nd orders are significantly weaker, with cross-CP components emerging at the ±1-st orders. This behavior is attributed to the simultaneous modulations of phase and intensity induced by the periodic splay nonlinear polarization grating (Fig. S5). For CP incident light, the SHG signal diffracts into three orders with distinct polarizations steered in a specific direction. Cross-CP components are observed at the zeroth and ±2-nd orders. Due to the weak intensity of SHG signals in the ±3-rd diffraction orders for all polarization incidences, they are not visible in Fig. 4 and are provided in the Supplementary Information (Fig. S6).

Figure 4d illustrates the nonlinear diffraction patterns of **Sample B**, which exhibits a spatially homogeneous orientation of polar LCs with $\theta =2{\rm{\pi }}/3$ (Fig. S7). Following polarization analysis (Fig. S8), we also demonstrate the capability to steer different polarization states into distinct diffraction orders. This implies that by adjusting the incident polarization, grating period, and splay angle, we can regulate the SHG signal’s diffraction behavior, including its diffraction orders, intensity distributions, and polarization states. As shown in Fig. 4b–d, both the diffraction angles (${\varnothing }_{{\rm{\omega }}}=2{\varnothing }_{2{\rm{\omega }}}$), intensity, and polarization distributions observed in our experiments are consistent with theoretical predictions. Our SHG measurements with differently polarized incident light confirm the diverse diffractive functionalities of FNLC devices, where the engineered space-varying Pancharatnam-Berry LCs impart distinct polarization and intensity modulations in the nonlinear optics realm, highlighting the interaction between the FW’s spin angular momentum and the nonlinearity Pancharatnam-Berry phase acquired, as described in Eq. (1) and (2).

### Dynamically programmed nonlinear Pancharatnam-Berry phase-controlled nonlinear diffractions

By leveraging the dynamic tunability of polar LC superstructures, we successfully demonstrate an active nonlinear diffractive beam steering capability. The space-varying Pancharatnam-Berry LC grating can be dynamically driven by applying an in-plane triangular-wave electric field with a frequency of 1 Hz and an alternating current voltage of 0.06 V·μm^-1^ (as shown in Supplementary Videos 2 and 3). As the electric field is applied and gradually increased, the splay angle of the polar LC grating transitions from its equilibrium state ($\theta ={\rm{\pi }}$, flat angle) to obtuse or acute angles depending on the sign of the electric field (Fig. 5a), enabling real-time tuning of the nonlinear Pancharatnam-Berry phase. The ion-doping ensures that this electrical modulation occurs smoothly and stably.

Figure 5b–d present the corresponding diffraction patterns of SHG signals and the intensity variations of the ±1-st and ±2-nd diffraction orders under different polarization states of incident light. For horizontally polarized light incidence, it has been observed that the zeroth diffraction order of the SHG signal exhibits an increasing trend in its intensity as the electric field changes from −0.06 V·μm^-1^ to +0.06 V·μm^-1^. Simultaneously, the intensities of the ±1-st and ±2-nd orders decrease, indicating a reduced diffraction efficiency with decreasing splay angles. Conversely, for vertically polarized light incidence, the intensity variations in different diffraction orders are small. By comparing with our simulations presented in Fig. 2c and Fig. S2, we find that the splay angle is adjusted to approximately ~$7{\rm{\pi }}/6$ and ~ $5{\rm{\pi }}/6$ when the electric field reaches its maximum value of 0.06 V·μm^-1^ (Fig. 5a). This result is consistent with the splay angle variation deduced from our texture observation. A slight deviation between the experimental results and simulated intensity variations in diffraction may be attributed to defects emerging during the application of the electric field and their impact on the overall nonlinear polarization. However, the general trend aligns with theoretical predictions. This demonstration highlights the potential of using dynamically tunable FNLC devices for active nonlinear photonic applications.

## Discussion

LCs represent a fascinating class of materials that can be engineered in three spatial dimensions. The implementation of ion-doped FNLCs enables the creation of large-area, defect-free polarization patterns, yielding millimeter-scale, high-quality nonlinear optical elements with spatially tailored distribution of the polar LCs (Fig. 3b). This approach guarantees robust and reproducible optical performance at the macroscopic scale, effectively overcoming defects that would otherwise disrupt desired light-matter interactions. By further manipulating the nonlinear LC system with three-dimensional controllable architectures, one can anticipate a host of novel phenomena and effects emerging from precisely engineered spatial ferroelectric structures. When polar LCs interact with light, they modulate not only the various degrees of freedom of the FW—phase, polarization, and amplitude—but also enable frequency conversion, thereby offering refined control over the resulting nonlinear phase, polarization, amplitude, and more.

A further key innovation in this work lies in the dynamic modulation of the polar LC orientations and hence the gradient Pancharatnam-Berry phase through very low external electric fields (0.06 V·μm^-1^). Since the director orientation responds predictably to the applied electric field, we achieve selective enhancement or suppression of specific diffraction orders from polar LC gratings, permitting real-time control over the spatial distribution of the SH signals. Rationally designed, structured electrodes (e.g., Fig. S9) could be further incorporated to enable pixelated in-plane switching of FNLCs and subsequently tailor the optical functionalities. This kind of dynamic control is highly desirable in future applications^13,58^ because it offers flexibility and adaptability. Instead of needing different physical components for different tasks, we could have a single component that adapts its function through applied voltages.

In conclusion, this study successfully demonstrates the feasibility of using ion-doped FNLCs as a platform for reconfigurable nonlinear Pancharatnam-Berry LC optics. By combining the inherent advantages of planar LC structures with the unique properties of ferroelectricity, we pave the way for next-generation nonlinear optical devices capable of advanced beam steering and signal processing. The dynamic tunability and versatility of our approach make it particularly promising for applications in quantum optics, adaptive optics, and nonlinear frequency conversion. Furthermore, the ability to achieve defect-free and large-scale (several cm^2^) polarization patterns ensures the scalability and reproducibility of our approach, providing a solid foundation for practical implementations in diverse fields.

## Materials and methods

### Materials and fabrications

Indium-tin-oxide glass substrates were first cleaned using UV-ozone treatment and then spin-coated with a photoalignment agent (SD1) at three sequential stages: 800 rpm for 10 s, followed by 3000 rpm for 40 s, and finally, 300 rpm for 1 s. The coated substrates were then cured at 100 °C for 10 min to ensure proper cross-linking of the photoalignment layer. Two such treated substrates were assembled into a cell using UV-curable adhesive, with the cell gap precisely measured as ~1 μm via interferometry.

To achieve spatially varying alignment configurations, a dynamic microlithography system based on a digital micromirror device was employed for multistep, partially overlapping exposure processes. The system utilized optimized exposure parameters, including intensity, duration, and sequence, to ensure high-quality pattern formation. After achieving the desired alignment pattern of SD1 with a total exposure dose of approximately 5 J·cm^-2^, the photoalignment layer demonstrated exceptional thermal stability throughout subsequent processing steps. It is important to note that prolonged UV illumination or exposure to high humidity should be avoided to prevent potential degradation of the photoalignment quality.

Following substrate preparation and assembly, the cell was infiltrated with FNLC RM734 doped with 0.92 wt% BMIM-PF6 at a temperature of 200 °C. A two-step thermal annealing process was then conducted to facilitate the self-assembly of FNLCs between the SD1 alignment layers. The first step involved rapid cooling from 190 °C to 130 °C at a rate of 5 °C·min^-1^. Then, the device was gradually cooled from 130 °C to 90 °C at a slower rate of 2 °C·min^-1^. This slow cooling ensured proper relaxation of flexible electrical couplings during phase transitions from the nematic phase through an intermediate phase (Nx) to the FN phase. Finally, the ferroelectric nematic polarization grating was formed under guidance from the photopatterned alignment layer, combined with flexoelectric coupling effects intrinsic to the FNLC material system.

### Characterizations

The resulting FNLC devices were characterized using a crossed-polarizing optical microscope (DM2700P, Leica) at various temperatures and conditions. These observations provided critical insights into the structural properties, response dynamics, and functionality of the fabricated devices.

In the nonlinear optical experiments, a Ti:sapphire femtosecond laser (Revolution, Coherent Inc.) was employed to pump an optical parametric amplifier. The system generated ultrashort pulses with a duration of approximately 40 fs at a repetition rate of 1 kHz. The collimated laser beam was focused onto the FNLC device using a spherical lens (*f* = 150 mm). After interaction with the FNLC structure, the generated SHWs were re-collimated through an objective lens with a numerical aperture of NA = 0.25. The polarization state of the resulting SHG diffraction spot was analyzed using a quarter-wave plate (AWP20Q-T4Q, JCOPTIX) and a linear polarizer, enabling detailed characterization of the nonlinear optical response.

## Supplementary information

Supplementary Information

Video 1

Video 2

Video 3

## Data Availability

The data that support the findings of this study are available within the paper and the supplementary material. Additional data related to this paper are available from the corresponding authors upon reasonable request.
