# Supplementary material for: Reconfigurable nonlinear Pancharatnam-Berry diffractive optics with photopatterned ferroelectric nematics
Source: Light Sci Appl. 2025 Sep 10;14:314. doi: 10.1038/s41377-025-01981-0 (PMC12423295; doi:10.1038/s41377-025-01981-0)
Supplement: Supplementary file 1 — Supplementary Information [file 41377_2025_1981_MOESM1_ESM.pdf]

Supplementary Information for

Reconfigurable nonlinear Pancharatnam-Berry diffractive optics with photopatterned ferroelectric nematics

Hui-Feng Chen[1], Xin-Yu Tao[1], Bo-Han Zhu[1,2], Jin-Tao Pan[1], Ling-Ling Ma[1,*], Chao Chen[1], Wen-Guo Zhu[3], Wei Chen[1,*], Yan-Qing Lu[1,*]

[1]National Laboratory of Solid State Microstructures, Key Laboratory of Intelligent Optical Sensing and Manipulation, College of Engineering and Applied Sciences, Nanjing University, Nanjing 210023, China.

[2]Research Institute of Superconductor Electronics (RISE), School of Electronic Science and Engineering, Nanjing University, Nanjing 210023, China.

[3]Key Laboratory of Optoelectronic Information and Sensing Technologies of Guangdong Higher Education Institutes, Department of Optoelectronic Engineering, Jinan University, Guangzhou 510632, China.

*Correspondence to malingling@nju.edu.cn (L.-L.M.); wchen@nju.edu.cn (W.C.); yqlu@nju.edu.cn (Y.-Q.L.)

Keywords: *liquid crystals, ferroelectric nematics, nonlinear Pancharatnam-Berry phase, light-matter interaction, nonlinear photonics*

Supplementary Text S1 Nonlinear polarization

Ferroelectric nematic liquid crystals (FNLCs), also known as polar LCs with broken inversion symmetry, exhibit strong nonlinear optical responses characterized by a non-zero second-order nonlinear optical susceptibility. At 1064 nm, the components of this susceptibility are given by $\chi_{aaa}^{(2)} = 11.2\ \mathrm{pm \cdot V^{-1}}$ and $\chi_{abb}^{(2)} = \chi_{bab}^{(2)} = \chi_{bba}^{(2)} = 1.2\ \mathrm{pm \cdot V^{-1}}$, where the *a*- and *b*-axes are parallel and perpendicular to the polar LC director, respectively[1]. To derive the nonlinear Pancharatnam-Berry phase, we recast the second-order susceptibility tensor $\chi_{ijk}^{(2)}$, where *i*, *j*, *k* = (*x*, *y*, *z*), on a circular polarization (CP) basis as $\chi_{lmn}^{(2)}$, where *l*, *m*, *n* = (*L*, *R*, *z*). The coordinate transformation is performed between the linear polarization basis and the CP basis:

$$\Lambda_{li} = \begin{pmatrix} 1 & \mathrm{i} & 0 \\ 1 & -\mathrm{i} & 0 \\ 0 & 0 & \sqrt{2} \end{pmatrix} / \sqrt{2}, \qquad \left[\Lambda^{-1}\right]_{il} = \begin{pmatrix} 1 & 1 & 0 \\ -\mathrm{i} & \mathrm{i} & 0 \\ 0 & 0 & \sqrt{2} \end{pmatrix} / \sqrt{2}. \tag{S1}$$

By coordinate transformation, the second-order susceptibility tensor in the CP basis yields:

$$\chi_{lmn}^{(2)} = \sum_{ijk} \Lambda_{li} \chi_{ijk}^{(2)} \left[\Lambda^{-1}\right]_{jm} \left[\Lambda^{-1}\right]_{kn}. \tag{S2}$$

As shown in Fig. 1a, when rotating the in-plane orientation of the LC director, the rotated nonlinear polarization will differ from the unrotated nonlinear polarization in the laboratory frame by an additional phase, which gives rise to the nonlinear Pancharatnam-Berry phase of the radiated second-harmonic (SH) signals. When the LC director is counterclockwise rotated by an angle α, the rotation matrices in the CP basis are given as

$$R_{l'l}(\alpha) = \begin{pmatrix} e^{-\mathrm{i}\alpha} & 0 & 0 \\ 0 & e^{\mathrm{i}\alpha} & 0 \\ 0 & 0 & 1 \end{pmatrix}, \qquad \left[R^{-1}(\alpha)\right]_{ll'} = \begin{pmatrix} e^{\mathrm{i}\alpha} & 0 & 0 \\ 0 & e^{-\mathrm{i}\alpha} & 0 \\ 0 & 0 & 1 \end{pmatrix}. \tag{S3}$$

Then, the nonlinear polarization[2,3] yields

$$P_l^{2\omega}(z) = \sum_{l} R_{ll'}(\alpha) P_{l'}^{2\omega}(z) = \varepsilon_0 \sum_{l'm'n'} R_{ll'}(\alpha) \chi_{l'm'n'}^{(2)} E_{m'}(z) E_{n'}(z). \tag{S4}$$

When substituting $E_{l'}^{\omega} = \sum_{l} R(\alpha)_{l'l} E_l^{\omega}$ into Eq. (S4), we obtain

$$P_l^{2\omega}(z) = \varepsilon_0 \sum_{mn} \sum_{l'm'n'} \left[R^{-1}(\alpha)\right]_{ll'} \chi_{l'm'n'}^{(2)} R(\alpha)_{n'n} R(\alpha)_{m'm} E_m(z) E_n(z). \tag{S5}$$

Consequently, the second-order susceptibility tensor of the FNLC molecule in the rotated CP basis in the laboratory frame can be written as

$$\chi_{lmn}^{(2)}=\sum_{l'm'n'}\left[R^{-1}(\alpha)\right]_{ll'}\chi_{l'm'n'}^{(2)}R_{n'n}(\alpha)R_{m'm}(\alpha). \tag{S6}$$

Therefore, the nonlinear Pancharatnam-Berry phase contributed by the transformation of coordinate system is

$$\left[R^{-1}(\alpha)\right]_{ll'}R_{n'n}(\alpha)R_{m'm}(\alpha). \tag{S7}$$

From Eq. (S6), we have components

$$\begin{array}{ll}\chi_{RRR}^{(2)}=\chi_{R'R'R'}^{(2)}\mathrm{e}^{\mathrm{i}\alpha} & \chi_{LLL}^{(2)}=\chi_{L'L'L'}^{(2)}\mathrm{e}^{-\mathrm{i}\alpha}\\ \chi_{RLL}^{(2)}=\chi_{R'L'L'}^{(2)}\mathrm{e}^{-\mathrm{i}3\alpha} & \chi_{LRR}^{(2)}=\chi_{L'R'R'}^{(2)}\mathrm{e}^{\mathrm{i}3\alpha}\\ \chi_{RRL}^{(2)}=\chi_{R'R'L'}^{(2)}\mathrm{e}^{-\mathrm{i}\alpha} & \chi_{LRL}^{(2)}=\chi_{L'R'L'}^{(2)}\mathrm{e}^{\mathrm{i}\alpha}\end{array}. \tag{S8}$$

where $\chi_{R'R'R'}^{(2)}=\chi_{R'L'L'}^{(2)}=\chi_{R'R'L'}^{(2)}=\chi_{L'L'L'}^{(2)}=\chi_{L'R'R'}^{(2)}=\chi_{L'R'L'}^{(2)}=\frac{1}{2\sqrt{2}}\chi_{aaa}^{(2)}$.

As illustrated in Fig. 1c, the polar LC film is divided into numerous infinitesimally thin layers along its thickness, with each layer having a thickness of dz. It is assumed that the orientation angle α remains unchanged across these layers. Assuming that the fundamental wave (FW) incident electric field is $\boldsymbol{E}_0^{\omega}$, to describe the linear optical process experienced by the FW as it propagates through a unit-thin LC layer, we have the Jones matrix

$$\boldsymbol{M}_{\mathrm{d}z}^{\omega}=\mathrm{e}^{-\mathrm{i}k\mathrm{d}zn_{\mathrm{eff}}}\begin{pmatrix}\cos(\mathrm{d}\Gamma_{\omega}/2) & -\mathrm{i}\sin(\mathrm{d}\Gamma_{\omega}/2)\mathrm{e}^{-\mathrm{i}2\sigma\alpha}\\ -\mathrm{i}\sin(\mathrm{d}\Gamma_{\omega}/2)\mathrm{e}^{\mathrm{i}2\sigma\alpha} & \cos(\mathrm{d}\Gamma_{\omega}/2)\end{pmatrix}, \tag{S9}$$

where, $\mathrm{d}\Gamma_{\omega}=\frac{2\pi}{\lambda}\left(n_{\mathrm{e}}-n_{\mathrm{o}}\right)\mathrm{d}z$ is the phase delay of FNLC, $n_{\mathrm{eff}}=(n_{\mathrm{o}}+n_{\mathrm{e}})/2$ is the effective index of light, n_{o}, n_{e} is the refractive index of ordinary and extraordinary light. For the Nth LC layer, the modulated FW at frequency ω is given by:

$$\boldsymbol{E}_{N\mathrm{d}z}^{\omega}=\left(\boldsymbol{M}_{\mathrm{d}z}^{\omega}\right)^{N}\cdot\boldsymbol{E}_0^{\omega}, \tag{S10}$$

The nonlinear polarization generated at this layer is:

$$\boldsymbol{P}_{N\mathrm{d}z}^{2\omega}=\boldsymbol{f}\left[\left(\boldsymbol{M}_{\mathrm{d}z}^{\omega}\right)^{N-1}\cdot\boldsymbol{E}_0^{\omega}\right],(N\geq 1). \tag{S11}$$

Here, $\boldsymbol{f}$ encapsulates the physical processes associated with nonlinear frequency conversion.

For an arbitrary polarized FW incident at normal incidence: $\boldsymbol{E}^{\omega}(0)=E_R^{\omega}(0)\hat{\mathbf{e}}_R+E_L^{\omega}(0)\hat{\mathbf{e}}_L$, the nonlinear polarization can be decomposed into right-handed and left-handed components, denoted as $\boldsymbol{P}_R^{2\omega}(N\mathrm{d}z)$ and $\boldsymbol{P}_L^{2\omega}(N\mathrm{d}z)$, respectively. These components are expressed as:

$$
\begin{aligned}
\boldsymbol{P}_R^{2\omega}(N\mathrm{d}z) &= \varepsilon_0 \begin{bmatrix} \chi^{(2)}_{R'R'R'}\mathrm{e}^{\mathrm{i}\alpha}\left(\boldsymbol{E}_R^{\omega}(N\mathrm{d}z)\right)^2 + \chi^{(2)}_{R'L'L'}\mathrm{e}^{-\mathrm{i}3\alpha}\left(\boldsymbol{E}_L^{\omega}(N\mathrm{d}z)\right)^2 \\ +2\chi^{(2)}_{R'R'L'}\mathrm{e}^{-\mathrm{i}\alpha}\left(\boldsymbol{E}_R^{\omega}(N\mathrm{d}z)\boldsymbol{E}_L^{\omega}(N\mathrm{d}z)\right) \end{bmatrix} \\
\boldsymbol{P}_L^{2\omega}(N\mathrm{d}z) &= \varepsilon_0 \begin{bmatrix} \chi^{(2)}_{L'R'R'}\mathrm{e}^{\mathrm{i}3\alpha}\left(\boldsymbol{E}_R^{\omega}(N\mathrm{d}z)\right)^2 + \chi^{(2)}_{L'L'L'}\mathrm{e}^{-\mathrm{i}\alpha}\left(\boldsymbol{E}_L^{\omega}(N\mathrm{d}z)\right)^2 \\ +2\chi^{(2)}_{L'R'L'}\mathrm{e}^{\mathrm{i}\alpha}\left(\boldsymbol{E}_R^{\omega}(N\mathrm{d}z)\boldsymbol{E}_L^{\omega}(N\mathrm{d}z)\right) \end{bmatrix}
\end{aligned}. \tag{S12}
$$

where $\boldsymbol{E}_R^{\omega}(N\mathrm{d}z)$ and $\boldsymbol{E}_L^{\omega}(N\mathrm{d}z)$ refer to the right-handed and left-handed CP components of propagating FW in the *z* plane, respectively.

For a simplified scenario, an incident FW with right-handed CP evolves into two distinct components $\boldsymbol{E}_R^{\omega}(N\mathrm{d}z) \propto \boldsymbol{E}_R^{\omega}(0)$ and $\boldsymbol{E}_L^{\omega}(N\mathrm{d}z) \propto \mathrm{e}^{\mathrm{i}2\alpha}\boldsymbol{E}_R^{\omega}(0)$ upon propagation and reaching the Nth LC layer due to the linear spin-coupling effect. By substituting them into Eq. (S12), the nonlinear polarization components at the Nth layer are derived as:

$$
\begin{aligned}
\boldsymbol{P}_R^{2\omega}(N\mathrm{d}z) &\propto \varepsilon_0\chi^{(2)}_{R'R'R'}\mathrm{e}^{\mathrm{i}\alpha}\left(\boldsymbol{E}_R^{\omega}(N\mathrm{d}z)\right)^2 \\
\boldsymbol{P}_L^{2\omega}(N\mathrm{d}z) &\propto \varepsilon_0\chi^{(2)}_{L'R'R'}\mathrm{e}^{\mathrm{i}3\alpha}\left(\boldsymbol{E}_R^{\omega}(N\mathrm{d}z)\right)^2
\end{aligned}. \tag{S13}
$$

The SH waves (SHWs) including the right-handed and left-handed CP components of each infinitesimal layer, which propagate towards the exit plane at *z* = *d* (*d* is the thickness of the FNLC film) and they are separately accumulated to obtain the output SH signal. Considering the propagation in the linear regime of the generated SHWs from *z* = *N*d*z* to *z* = *d*, we obtain the output nonlinear polarization of Nth LC layer as

$$
\boldsymbol{P}_N^{2\omega}(z=d) = \boldsymbol{M}_{\mathrm{d}z}^{2\omega}\boldsymbol{P}_{(N-1)\mathrm{d}z}^{2\omega} + \boldsymbol{P}_{N\mathrm{d}z}^{2\omega}, (N \geq 1), \tag{S14}
$$

where former item $\boldsymbol{M}_{\mathrm{d}z}^{2\omega}\boldsymbol{P}_{(N-1)\mathrm{d}z}^{2\omega}$ is the SHWs which has been generated and propagated from first layer to the $(N\text{-}1)^{\text{th}}$ layer, latter item $\boldsymbol{P}_{N\mathrm{d}z}^{2\omega}$ is the SHWs generated by the $(N)^{\text{th}}$ layer. The emergence of distinct nonlinear Pancharatnam-Berry phases from the entire FNLC is independent of the propagation process, hence, when these SHWs propagate and reach the exit plane, we obtain $\begin{matrix} \boldsymbol{P}_R^{2\omega}(d) \propto \mathrm{e}^{\mathrm{i}\alpha} \\ \boldsymbol{P}_L^{2\omega}(d) \propto \mathrm{e}^{\mathrm{i}3\alpha} \end{matrix}$. The output nonlinear polarization fields are summed separately for each infinitesimal layer, and the result is

$$
P_{\mathrm{sum}}^{2\omega}(z=d) = \sum_{N}^{1,2,\cdots,d/\mathrm{d}z} P_N^{2\omega}(z=d). \tag{S15}
$$

Supplementary Figures:

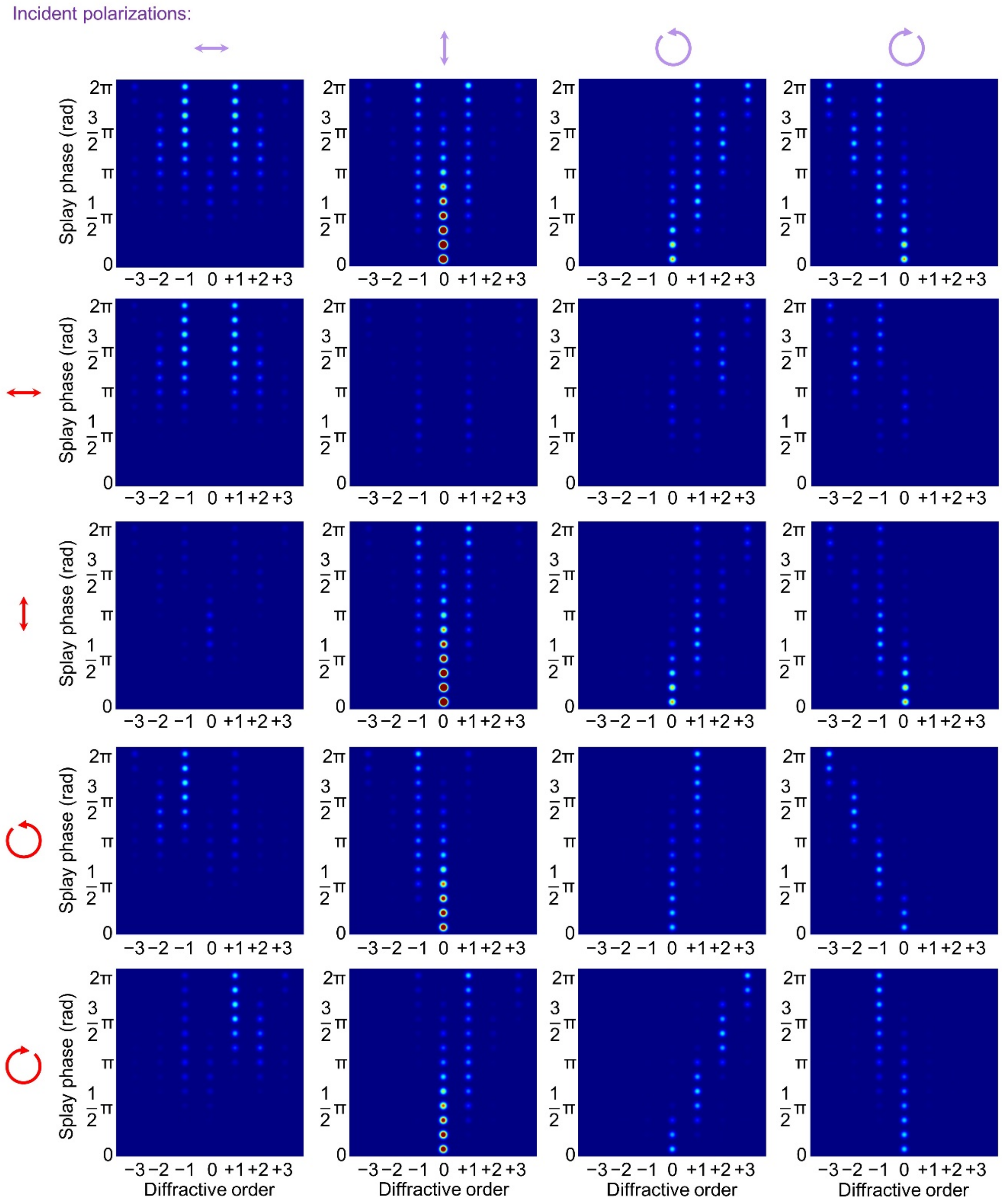

Fig. S1 | Simulated diffraction patterns and their polarization components of SH signals for FW with horizontal polarization, vertical polarization, left-handed CP, and right-handed CP incidences.

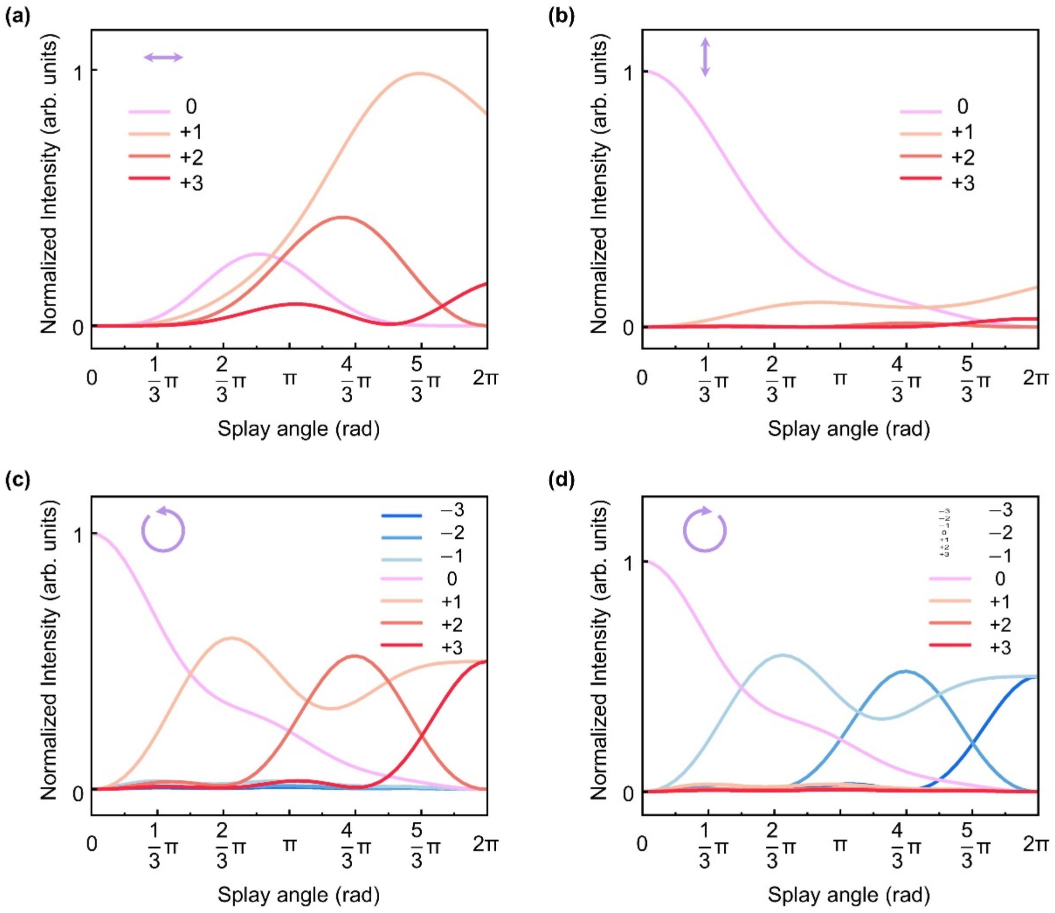

Fig. S2 | Intensity variations in different diffraction orders under illumination with horizontally polarized (a), vertically polarized (b), left-handed CP (c), and right-handed CP light (d).

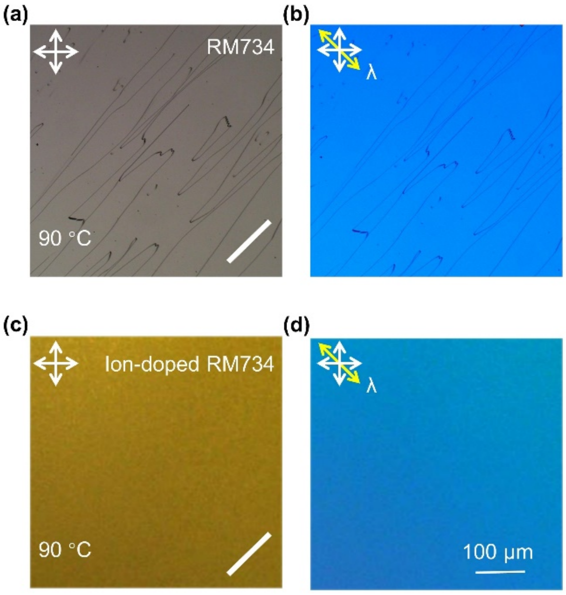

Fig. S3 | Polarizing optical microscope images of pure FNLCs and ion-doped FNLCs. (a) Texture of pure RM734 under the crossed polarizing optical microscope. White lines: alignment direction. (b) Texture of pure RM734 under the cross-polarizing optical microscope with a full wave plate (yellow arrow) insertion. (c) Texture of ion-doped RM734 under the crossed polarizing optical microscope. (b) Texture of ion-doped RM734 under the cross-polarizing optical microscope with a full wave plate (yellow arrow) insertion.

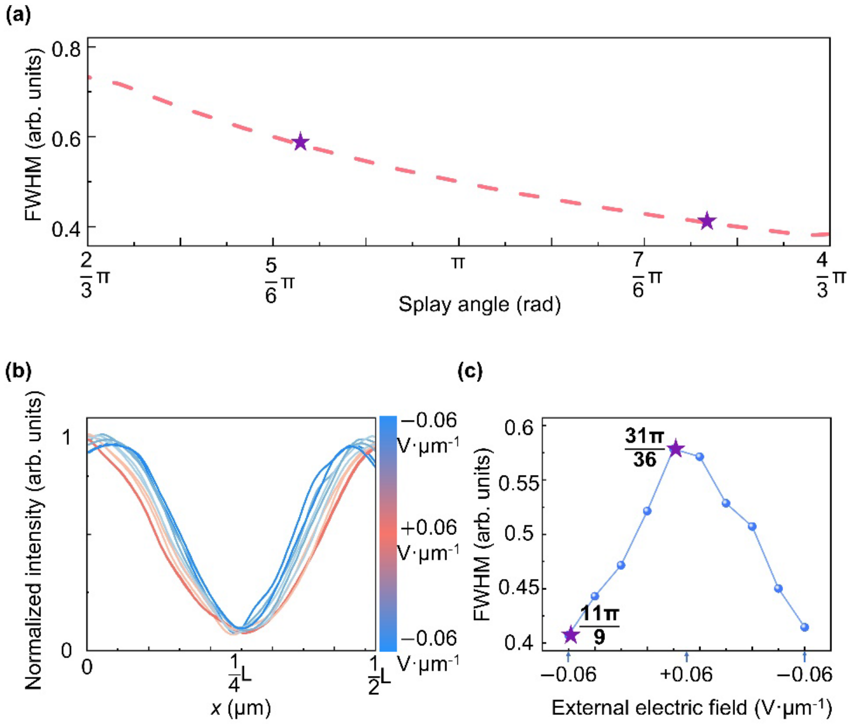

Fig. S4 | Voltage-dependent modulation of the splay angle in polar LC grating. (a) Simulated relationship between the splay angle and the full width at half maximum (FWHM). (b) Intensity profiles extracted from the cross-polarized optical microscopy images of the polar LC grating, showing dynamic evolution under a cycle voltage waveform. (c) Experimental FWHM variation as a function of applied voltage.

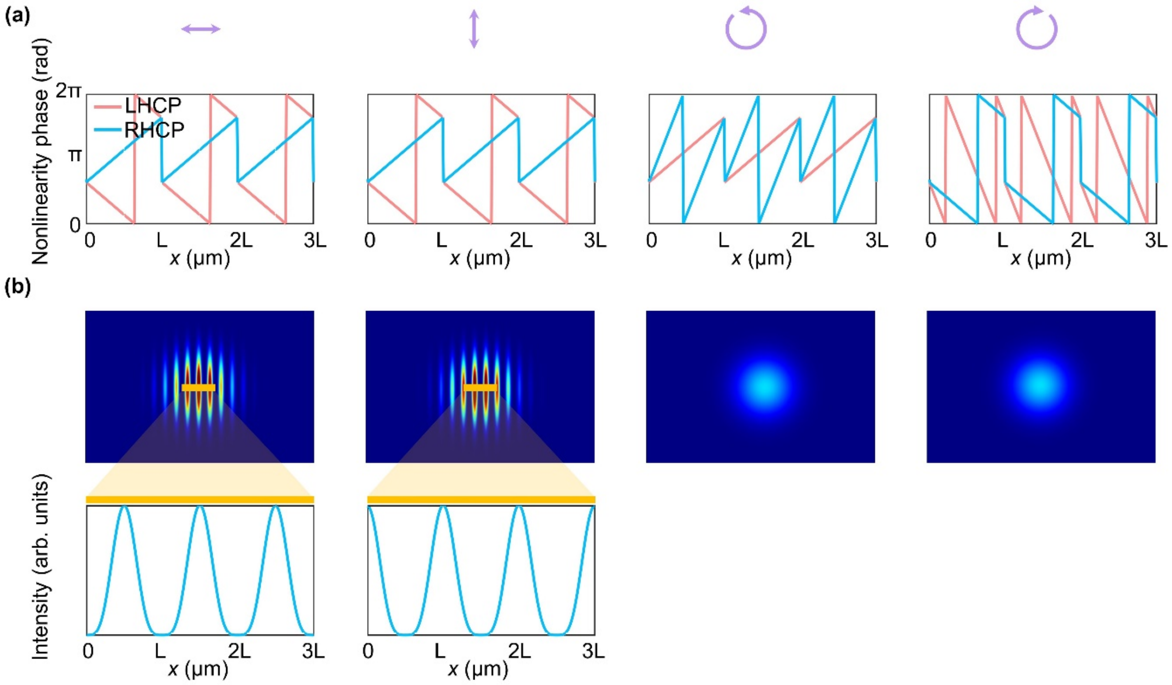

Fig. S5 | Simulated nonlinearity phases (a) and intensities (b) excited from the polar LC grating with the splay angle of **π** under illumination with horizontally polarized, vertically polarized, left-handed CP, and right-handed CP light.

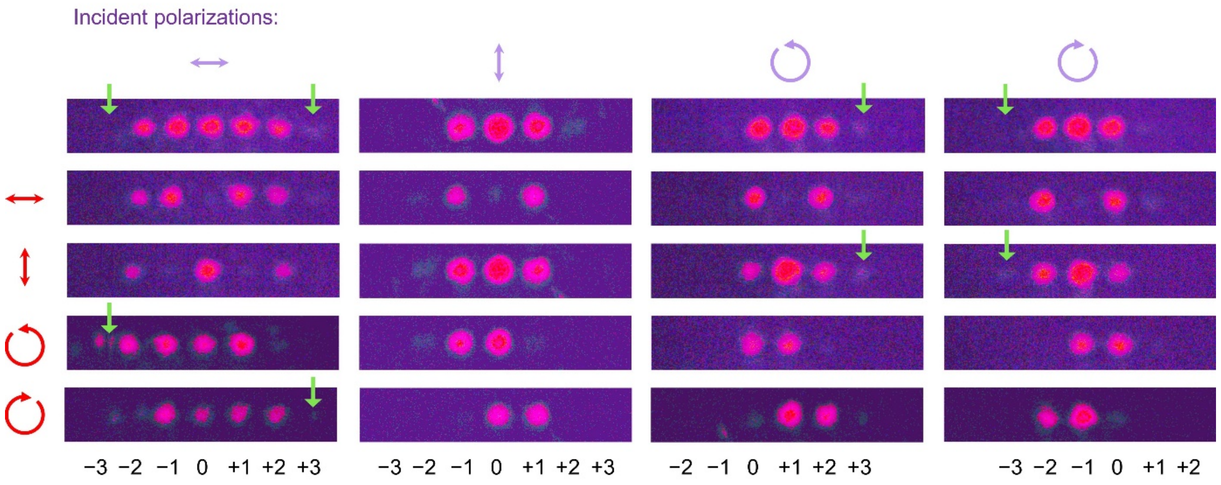

Fig. S6 | Saturated diffraction patterns in Figure 4c. Faint third-order diffraction features are indicated by green arrows.

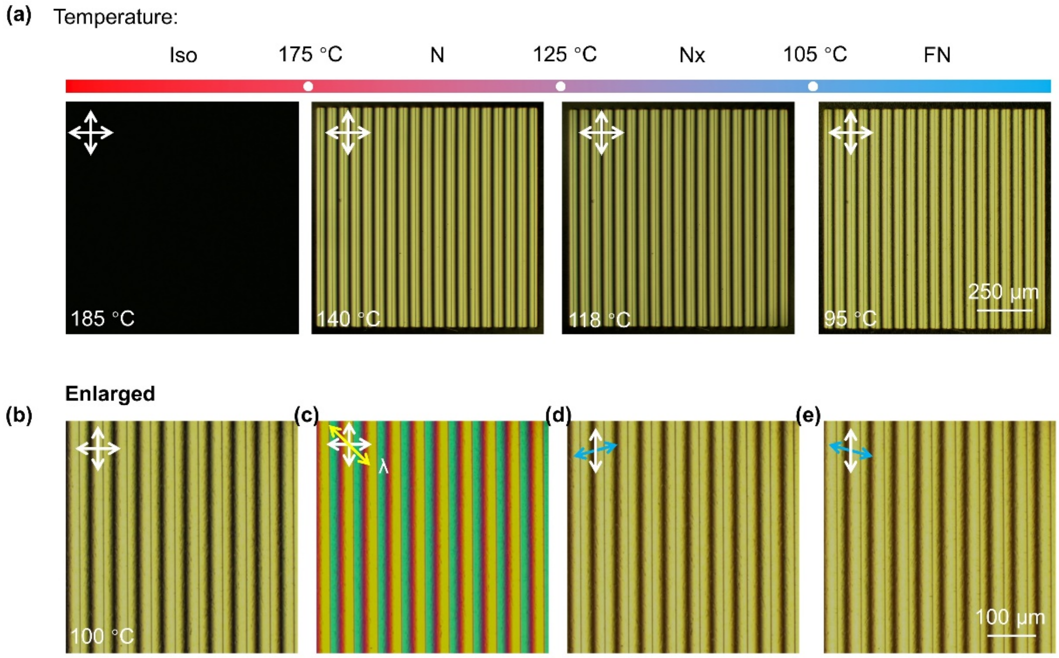

Fig. S7 | Gradient nonlinear Pancharatnam-Berry LC devices with the splay angle of $\mathbf{2\pi/3}$. (a) Polarizing optical microscope images of defect-free gradient polarity patterns of ion-doped FNLCs during the thermal annealing process. Scale bar: 250 μm. Iso: isotropic phase; N: nematic phase; Nx: mesophase; FN: ferroelectric nematic phase. (b-e) Polarizing optical microscope characterizations of the polar LC arrangement in the device. These images include (b) an enlarged texture, (c) an image under the cross-polarizing optical microscope with a full wave plate insertion, (d, e) two images of rotating the analyzer in opposite directions with the included angles of 75° and 105° between the polarizer and analyzer. Scale bar: 100 μm.

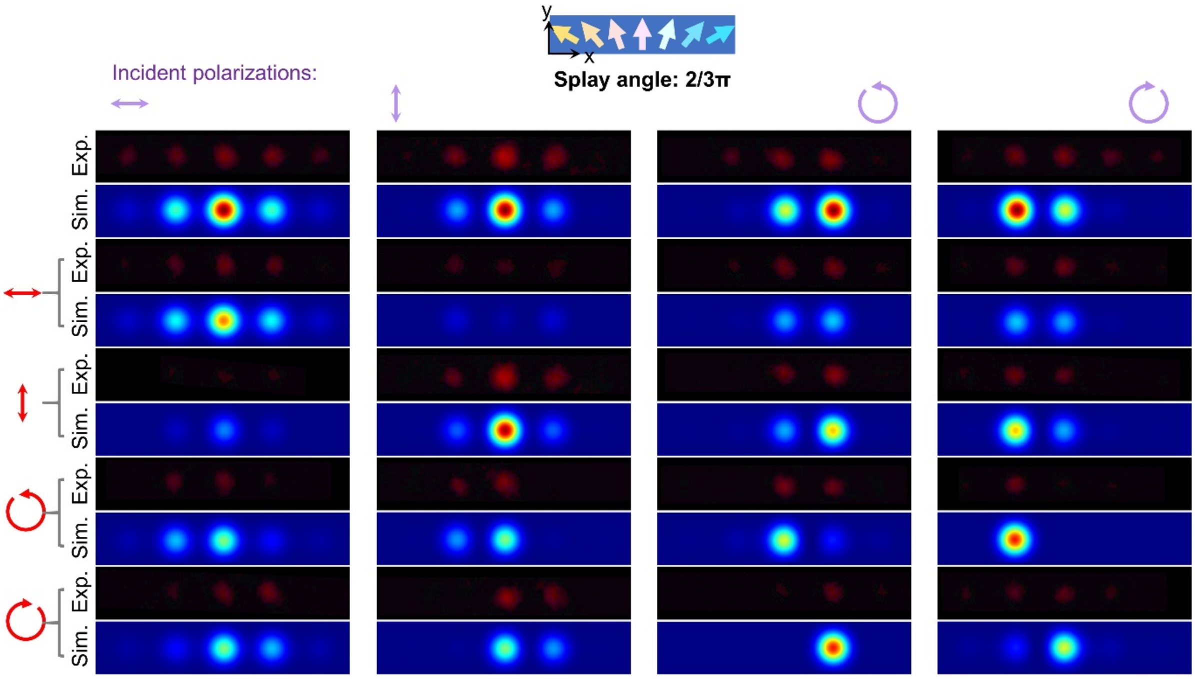

Fig. S8 | Simulated and experimental nonlinear diffraction patterns of the FNLC device with a splay angle of $\mathbf{2\pi/3}$, along with their polarization components of SH signals under different polarization states of the FW, including horizontally polarized, vertically polarized, left-handed CP, and right-handed CP incidences. Above, the colored unidirectional arrows depict the orientation of the polar LC molecules.

(a)

(b)

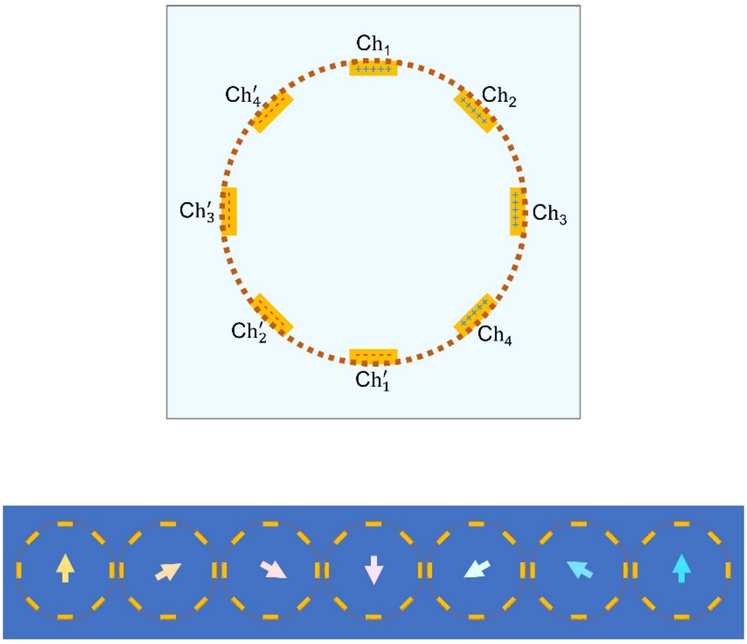

Fig. S9 | Electrically switching scheme. (a) Structured electrodes, with four sets of positive and negative electrodes, used to apply sequential driving voltage. Ch: Channel. (b) Pixelated in-plane switching of FNLCs, with an example of achieving a splay angle of $\mathbf{2\pi}$.

Table 1: Preset driving condition

Number	1	2	3	4	5	6	7	8	9	10
**Cycles**	>10	>10	>10	>10	>10	>10	>10	>10	>10	>10
**Waveform**	Tri.	Sin.	Sin.	DC	DC	Squ.	Sin.	Sin.	Tri.	Tri.
**Frequency (Hz)**	0.5	0.5	1			1	1	0.5	0.5	1
**Voltage (V·μm^{-1})**	0.06	0.06	0.06	0.06	0.06	0.012	0.06	0.06	0.06	0.06

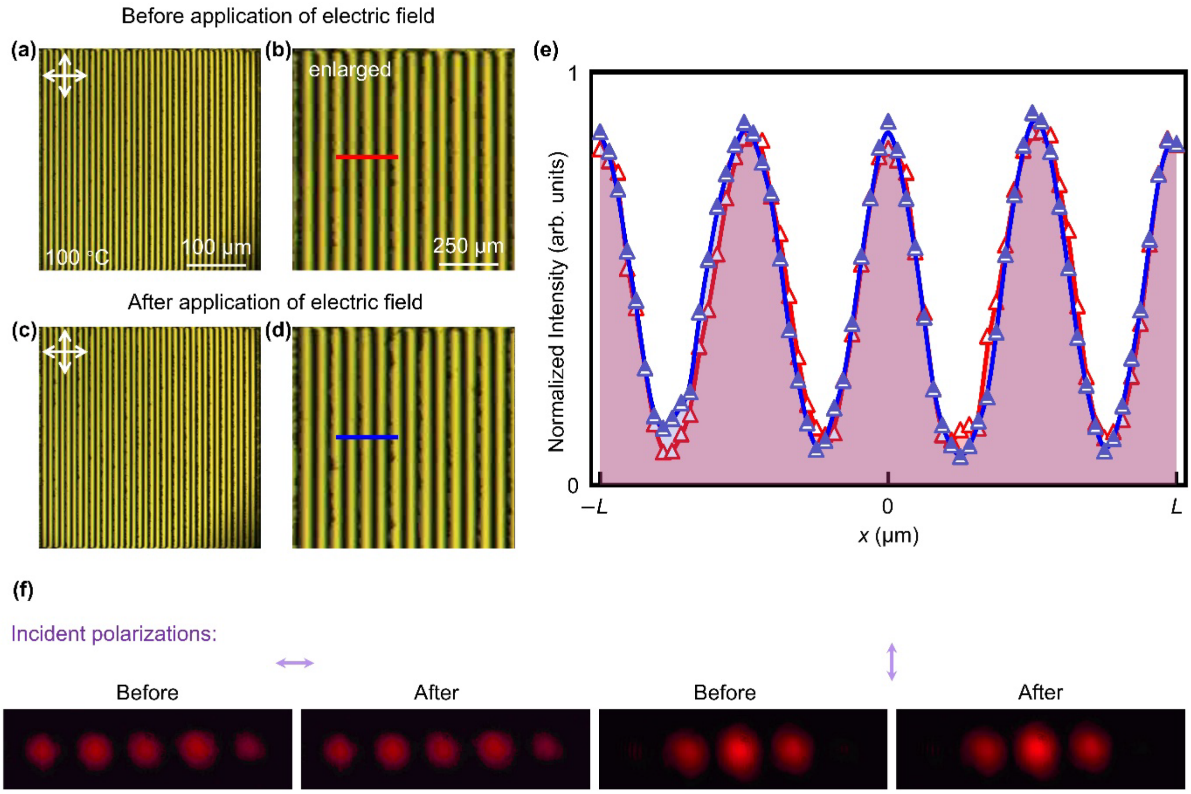

Fig. S10 | Patterned FNLC grating textures and diffraction patterns under electrical stimulation. The figure presents the results of 10 experimental measurements, with each measurement conducted over multiple (>10) electrical stimulation cycles. **Table 1** details the preset voltage conditions for these measurements. **(a-d)** Crossed polarizing optical microscope images of the patterned FNLC grating texture before and after the application of the electric field (i.e., after 10 experimental measurements), with (b) and (d) showing the corresponding enlarged view. **(e)** Intensity profiles derived from quantitative grayscale analysis of the patterned FNLC grating textures before and after the electric field application. **(f)** Second-harmonic generation diffraction patterns observed before and after multiple electrical stimulation cycles, excited by horizontally and vertically polarized FWs.

Here, we present experimental measurements of the patterned FNLC grating subjected to electrical stimulations, with each measurements repeated multiple times (>10) (Table 1). The initial and final textures exhibit significant consistency (Figs. S10a and S10c), as evidenced by local magnifications (Figs. S10b and S10d) and quantitative grayscale analysis of the textures (Fig. S10e). This indicates that an appropriately configured electric field does not induce noticeable damage to the polar liquid crystal structure. Furthermore, the second-harmonic generation diffraction patterns and intensities, excited by horizontally and vertically polarized fundamental waves, remain consistent before and after the electrical stimulation experiments (Fig. S10f). This confirms that the functionality and SH efficiency of the patterned FNLCs are preserved under appropriate electrical stimulation.

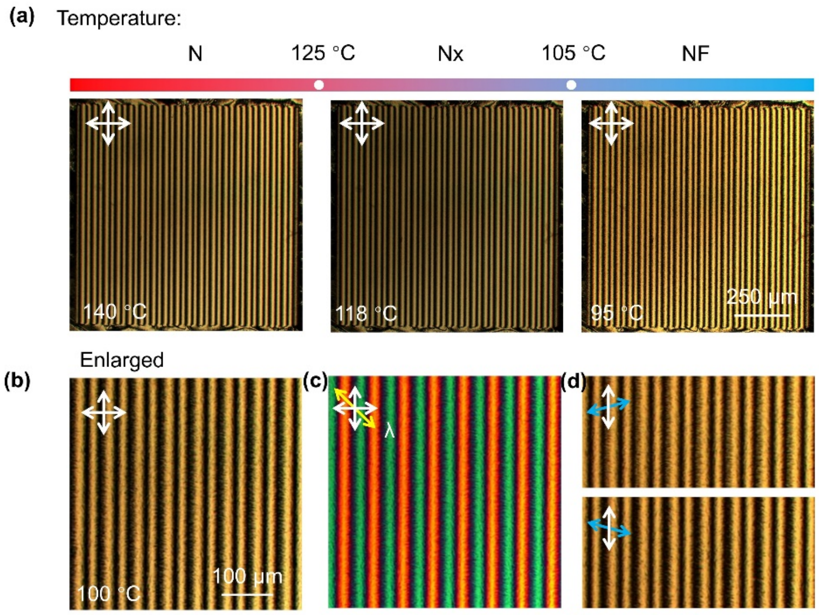

Fig. S11 | Reheating of the reconfigurable nonlinear Pancharatnam-Berry LC device with a splay angle of π after ~3 months of storage. This study examines the same sample (Sample A) utilized in the main text Figs. 3, 4, and 5 once after fabrication. **(a)** Polarizing optical microscope images depict defect-free periodic polarity patterns of ion-doped FNLCs during the thermal annealing process. Scale bar: 250 μm. **(b-d)** Polarizing optical microscope characterizations of the polar LC arrangement in the device include: (b) an enlarged texture, (c) an image under crossed polarizers with a full-wave plate insertion, and (d) two images obtained by rotating the analyzer in opposite directions, with included angles of 75° and 105° between the polarizer and analyzer. Scale bar: 100 μm.

These observations demonstrate that the underlying polar LC director structure remains stable after 3 months of storage, confirming the robustness of the device.

Supplementary Movie

Movie S1. Electrically tuning of the splay angle of the polar LC grating. The initial splay angle is π. An in-plane electric field of a symmetrical triangular wave (1 Hz, 0.06 $V \cdot \mu m^{-1}$) is applied to the device.

Movie S2. Dynamic modulation of the diffraction pattern under illumination with horizontally polarized light. An in-plane electric field of a symmetrical triangular wave (1 Hz, 0.06 $V \cdot \mu m^{-1}$) is applied to the device.

Movie S3. Dynamic modulation of the diffraction pattern under illumination with vertically polarized light. An in-plane electric field of a symmetrical triangular wave (1 Hz, 0.06 $V \cdot \mu m^{-1}$) is applied to the device.

References

1 Folcia, C. L., Ortega, J., Vidal, R., Sierra, T. & Etxebarria, J. The ferroelectric nematic phase: an optimum liquid crystal candidate for nonlinear optics. *Liq. Cryst.* **49**, 899-906 (2022).

2 Boyd, R. W. *Nonlinear optics*, 3rd edn edn. (Academic Press, 2008).

3 Pan, J.-T. *et al.* Nonlinear geometric phase coded ferroelectric nematic fluids for nonlinear soft-matter photonics. *Nat. Commun.* **15**, 8732 (2024).
